# Association between continuous glucose monitoring-derived glycemic control indices and urinary biomarkers of diabetic kidney disease: Hyogo Diabetes Hypoglycemia Cognition Complications study

**DOI:** 10.1007/s00592-023-02214-9

**Published:** 2023-11-25

**Authors:** Ayako Takagi, Yoshiki Kusunoki, Mana Ohigashi, Keiko Osugi, Chikako Inoue, Maki Inoue, Taku Tsunoda, Manabu Kadoya, Kosuke Konishi, Tomoyuki Katsuno, Hidenori Koyama, Hyogo Diabetes Hypoglycemia Cognition Complications (HDHCC) study group

**Affiliations:** 1 Department of Diabetes, Endocrinology and Clinical Immunology, School of Medicine, Hyogo Medical University, 1-1, Mukogawa-cho, Nishinomiya, Hyogo 663-8501 Japan; 2 Department of Occupational Therapy, School of Rehabilitation, Hyogo Medical University, Nishinomiya, Hyogo Japan

**Keywords:** Continuous glucose monitoring, Time in range, Glycemia risk index, Urinary *N*-acetyl-β-d-glucosaminidase, Urinary albumin

## Abstract

**Aims:**

Glomerular damage and proximal tubular damage play an important role in the pathogenesis of diabetic kidney disease. This study aimed to investigate the relationship between the urinary markers of proximal tubular injury, including urinary liver-type fatty acid-binding protein-to-creatinine ratio (uL-FABP/Cr) and urinary *N*-acetyl-β-d-glucosaminidase-to-creatinine ratio (uNAG/Cr), and glycemic control status.

**Methods:**

This cross-sectional study included 245 and 39 patients with type 2 diabetes mellitus (T2DM) and non-T2DM (NDM), respectively. The participants of this study were fitted with retrospective CGM, and glycemic control indices, such as time in range (TIR) and glycemia risk index (GRI), were calculated.

**Results:**

The results were presented as medians (interquartile ranges). The uL-FABP/Cr was significantly higher in the microalbuminuria than in the normo-albuminuria group [4.2 (2.7–7.1) and 2.2 (1.4–3.4) μg/gCr, respectively, *P* < 0.001], while the uNAG/Cr in the normo-albuminuria group [6.3 (4.5–10.1) U/gCr] was significantly higher than that in the NDM group [5.3 (3.8–6.3) U/gCr, *P* = 0.048] but significantly lower than that in the microalbuminuria group [9.2 (6.4–11.1) U/gCr, *P* = 0.004]. The multivariate logistic regression analysis indicated that CGM-derived TIR was significantly associated with the urinary albumin-to-creatinine ratio [uAlb/Cr, odds ratio (OR) 0.985, 95% confidence interval (CI) 0.971–0.998, *P* = 0.029] and uNAG/Cr (OR 0.973, 95% CI 0.957–0.989, *P* = 0.001) independent of renal function. GRI was similarly associated with uAlb/Cr and uNAG/Cr.

**Conclusion:**

The findings of this study indicated that uNAG/Cr was elevated before albuminuria development and was associated with CGM-derived TIR and GRI.

## Introduction

Diabetic nephropathy (DN) is one of the microvascular complications of diabetes mellitus (DM) and a major cause of end-stage renal failure [1–4]. The number of patients with DM is increasing worldwide, and the prevention of DM onset and progression has become critical clinical issues [1–4]. The appearance of microalbuminuria precedes the decline in the estimated glomerular filtration rate (eGFR) in typical DN [3]. Microalbuminuria is associated with the loss of glomerular charge selectivity, and the urinary albumin-to-creatinine ratio (uAlb/Cr) is used as an early marker of DN and glomerular hyperfiltration [4, 5]. Therefore, the uAlb/Cr and eGFR have been used as measures to evaluate DN in numerous clinical trials [4]. However, many patients with DM and renal dysfunction did not have albuminuria [2, 3, 6]. Against this background, the disease concept for DN has changed, and the term diabetic kidney disease (DKD) is now widely used [7].

Many reports have suggested that in addition to glomerular hyperfiltration and glomerular hemodynamic disturbances, proximal tubular damage plays an important role in the pathogenesis of DKD [8–10]. Urinary markers of tubular injury include urinary liver-type fatty acid-binding protein-to-creatinine ratio (uL-FABP/Cr) and urinary *N*-acetyl-β-d-glucosaminidase-to-creatinine ratio (uNAG/Cr) [11]. These urinary biomarkers have been reported to be useful for the early diagnosis of DKD onset, and their excretion rate increases with DKD progression [11–18].

Previous studies have reported that glycemic variability indices and time in range (TIR) assessed by continuous glucose monitoring (CGM) are associated with uAlb/Cr [19, 20]. However, it has been reported that uAlb/Cr is not elevated in many DKD cases, and urinary tubular injury markers are useful in predicting DKD onset [8–10]. Owing to the association between longer TIRs and a reduced risk of diabetes complications [21, 22] and their superior accuracy in assessing hypoglycemic and hyperglycemic status compared with the traditional measure, HbA1c [23, 24], recent international consensus has underscored the significance of TIR in DM management [25]. Nonetheless, few studies have investigated the association between CGM-derived glycemic control indices and markers of tubular damage. Therefore, this study aimed to investigate the association between CGM-derived glycemic control indices such as TIR and GRI and uAlb/Cr used as a marker of glomerular damage, and uL-FABP/Cr and uNAG/Cr used as markers of tubular injury in patients with type 2 diabetes mellitus (T2DM) and non-diabetes mellitus (NDM) cases.

## Methods

### Study design and subjects

The present study was conducted as part of the Hyogo Diabetes Hypoglycemia Cognition Complications (HDHCC) study. The HDHCC study is a multicenter cohort study that investigated the relationship between glycemic control status and chronic vascular complications in patients with at least one cardiovascular risk factor, including diabetes, hypertension, dyslipidemia, and obesity, who visited outpatient clinics. This study included T2DM and NDM subjects aged 40 and 80 years who completed CGM and urinalysis at Hyogo Medical University Hospital between April 2018 and January 2023. The diagnosis of T2DM was made based on the guidelines of the Japan Diabetes Society [26]. In short, the diabetologists diagnosed T2DM for individuals with a fasting plasma glucose of  ≥ 126 mg/dL, causal plasma glucose of  ≥ 200 mg/dL, 2-h plasma glucose of  ≥ 200 mg/dL during a 75-g oral glucose tolerance test, or HbA1c of ≥ 6.5% and those who had been diagnosed with T2DM or those who were on diabetes medications [26]. Moreover, the diabetologists diagnosed NDM for participants in the HDHCC study who did not meet any of the aforementioned diagnostic criteria for T2DM [26].

The exclusion criteria were as follows: (i) those with type 1 DM, (ii) those with nephrotic syndrome (proteinuria of  ≥ 3.5 g/gCr at spot urine) or chronic renal failure (eGFR of  < 30 mL/min/1.73 m^2^) [27], (iii) those with severe hepatic dysfunction (defined as alanine transaminase  ≥ 3 times the upper limit of normal), (iv) those in whom CGM data could not be obtained for >7 consecutive days, (v) those who underwent renal transplantation, and (vi) those deemed ineligible for this study by their physician.

### Evaluation of DKD

A urinalysis was performed in the morning on the same day as the CGM was worn. Urinary albumin was measured with an immunoturbidimetric method using Autowaco Microalbumin (Fujifilm Wako Pure Chemicals Corp., Osaka, Japan). Urinary creatinine was measured with an enzymatic method using Cygnus Auto CRE (Sino-Test Corp., Tokyo, Japan). In addition, uL-FABP was measured with the chemiluminescent enzyme immunoassay method using Lumipulse Presto L-FABP (Fujirebio Co., Ltd., Tokyo, Japan), and uNAG was measured with the colorimetric method using L-Type Wako NAG (FUJIFILM Wako Pure Chemical Corp., Osaka, Japan). The lower limit of measurement was 3 μg/mL for urinary albumin, 0.5 ng/mL for uL-FABP, and 0.4 U/L for uNAG. If the measured value was less than the lower limit of measurement, the lower limit of measurement/Cr was substituted.

#### CGM

Participants were instructed to wear FreeStyle Libre Pro^®^ (Abbott Japan, Tokyo, Japan) as a CGM for 14 days. All sensor glucose data from the day after CGM attachment were analyzed according to the consensus statement of CGM and metrics for clinical trials [25]. Then, TIR (percentage of time spent in the consensus target glucose range of 70–180 mg/dL), time in the tight range (percentage of time spent in the target glucose range 70–140 mg/dL, TIT), time above range (percentage of time spent with  > 180 mg/dL, TAR), time below range (percentage of time spent with  < 70 mg/dL, TBR), coefficient of variation (CV), standard deviation (SD) of mean glucose, and glucose management indicator (GMI) were calculated according to the consensus report [25]. The glycemia risk index (GRI) was calculated using the following formula based on a previous report [28].

GRI = (3.0 × TBR^<54 mg/dL^) + (2.4 × TBR^54−69 mg/dL^) + (1.6 × TAR^>250 mg/dL^) + (0.8 × TAR^181−250 mg/dL^)

(Note that the upper limit of GRI was set at 100.)

### Other parameters

Blood samples were taken at the time of wearing the CGM device. HbA1c was measured with high-performance liquid chromatography using HLC-723G11 (Tosoh Corporation, Tokyo, Japan). Serum creatinine levels were measured with an enzymatic method using Cygnus Auto CRE (Sino-Test Corp., Tokyo, Japan), and serum cystatin C levels were measured with the gold colloid agglutination method using Nescort GC Cystatin C (Nm) (Alfresa Pharma Corp., Osaka, Japan). The eGFR and cystatin C with eGFR (eGFRcys) for each participant were calculated from the serum creatinine and serum cystatin C levels based on previous reports [29, 30].

We defined dyslipidemia as a low-density lipoprotein cholesterol level of ≥ 140 mg/dL, high-density lipoprotein cholesterol level of < 40 mg/dL, and triglyceride level of  ≥ 150 mg/dL or receiving treatment for dyslipidemia. Hypertension was defined as a systolic blood pressure of  ≥ 140 mmHg, diastolic blood pressure of  ≥ 90 mmHg, or receiving treatment for hypertension.

### Statistical analysis

The results were presented as medians (interquartile ranges) unless otherwise stated. Patients with T2DM were divided into three groups based on uAlb/Cr values: normo-albuminuria (< 30.0 mg/gCr, normo), microalbuminuria (30.0–299.9 mg/gCr, micro), and macroalbuminuria (≥ 300.0 mg/gCr, macro) groups. Then, the normo group was used as a control and compared with the micro, macro, and NDM groups. Then, patients with T2DM were divided into two groups: TIR  < 70.0% (low-TIR group) and TIR  ≥ 70.0% (high-TIR group). The high-TIR group was used as a control and compared with the low-TIR and NDM groups. The Kruskal–Wallis and Steel tests were used to determine differences in quantitative data, and the chi-squared test was used to find differences in qualitative data between the groups.

The ordinal logistic regression analysis investigated the association between albuminuria categories and each glycemic control index. In Model 1, ordinal logistic regression analysis was performed with stages of albuminuria as the objective variable and each glycemic control index as explanatory variables, adjusted for duration of diabetes, sex, age, body mass index (BMI), smoking status, and presence of hypertension and dyslipidemia. Model 2 was adjusted for eGFR in addition to the factors in Model 1. Based on previous reports, a uL-FABP/Cr of  > 8.4 μg/gCr as uL-FABP/Cr positive and a uNAG/Cr of  > 5.8 U/gCr as uNAG/Cr positive [12, 31]. Binary logistic regression analysis was performed for tubular injury markers as objective variables and each glycemic control index as explanatory variables. Model 1 was adjusted for duration of diabetes, sex, age, BMI, smoking status, and presence of hypertension and dyslipidemia. Model 2 was adjusted for eGFR and uAlb/Cr in addition to the factors in model 1.

Statistical analyses were conducted using the BellCurve software version 4.05 (Social Survey Research Information Co., Ltd., Tokyo, Japan), with *P* < 0.05 indicating statistical significance.

## Results

### Participant background

The characteristics of the participants are shown in Table 1. The study enrolled 284 participants, consisting of 245 patients with T2DM and 39 cases with NDM. As comorbidities, 69.0% and 80.0% of the participants with T2DM had hypertension and dyslipidemia, respectively. In the NDM group, 74.4% had hypertension, while 79.5% had dyslipidemia. The NDM group was significantly older than the normo group [71 (68–74) vs*.* 68 (62–72) years old, *P* = 0.010]. In addition, the NDM group had significantly lower HbA1c levels but significantly higher TIR compared with the normo group (all *P* < 0.001).

**Table 1:** Characteristics of the study participants

	T2DM			NDM (*N* = 39)	*P*
	Normo-albuminuria (*N* = 166)	Microalbuminuria (*N* = 58)	Macroalbuminuria (*N* = 21)		
Male, %	67.5	67.2	76.2	46.2	0.050
Age, years	68 (62–72)	68 (62–72)	71 (65–74)	71 (68–74)*	0.008
Duration of T2DM, years	11.5 (5.3–21.0)	12.5 (9.3–24.0)	17.0 (9.0–24.0)	0 (0–0)**	< 0.001
BMI, kg/m^2^	24.0 (21.9–25.7)	24.4 (23.0–27.7)	24.9 (22.4–27.6)	23.9 (22.1–26.1)	0.185
SBP, mmHg	126.0 (115.0–137.0)	129.0 (118.3–140.8)	138.0 (130.0–146.0)*	130.5 (121.5–139.0)	0.027
DBP, mmHg	75.0 (68.0–82.0)	78.0 (71.0–83.0)	75.0 (70.0–92.0)	78.0 (70.5–83.0)	0.319
UA, mg/dL	5.2 (4.4–6.1)	5.2 (4.5–6.1)	5.6 (5.2–6.7)	5.2 (4.8–6.1)	0.183
HbA1c, % [mmol/mol]	7.0 (6.6–7.5) [52]	7.2 (6.8–7.7) [55]	7.1 (6.4–7.8) [54]	5.9 (5.7–6.1) [40]**	< 0.001
GMI, %	6.7 (6.2–7.1)	6.8 (6.3–7.3)	7.0 (6.2–8.0)	5.9 (5.7–6.0)**	< 0.001
TIR, %	78.2 (69.2–87.6)	77.8 (61.6–88.2)	73.9 (41.7–87.4)	93.8 (91.1–97.4)**	< 0.001
TAR, %	17.7 (8.2–29.0)	20.2 (7.2–36.8)	22.8 (7.0–57.4)	0.9 (0.2–4.5)**	< 0.001
TBR, %	0.2 (0.0–2.3)	0.5 (0.0–2.4)	1.1 (0.0–3.4)	2.5 (0.9–5.3)**	< 0.001
GRI, %	21.5 (12.5–39.1)	24.8 (13.3–40.9)	25.1 (19.8–64.2)	9.6 (4.7–13.7)**	< 0.001
Current smoker, %	17.5	19.0	38.1	5.1	0.016
Hypertension, %	60.8	81.0	71.4	74.4	0.027
Dyslipidemia, %	77.1	86.2	85.7	79.5	0.442
T-chol, mg/dL	176.5 (154.0–203.8)	181.0 (151.3–198.8)	194.0 (166.0–220.0)	188.0 (174.0–200.5)	0.145
HDL-chol, mg/dL	58.5 (48.0–70.0)	53.5 (44.0–65.8)	52.0 (45.0–65.0)	60.0 (53.0–71.0)	0.108
CCBs, %	27.7	56.9	90.5	48.7	< 0.001
RAS inhibitors, %	38.6	62.1	95.2	28.2	< 0.001
Statins, %	54.2	63.8	61.9	61.5	0.553
SGLT2 inhibitors, %	22.9	41.4	38.1	0	< 0.001
GLP-1 RAs, %	9.6	22.4	19.0	0	0.004
Other T2DM medicines, %	78.3	89.7	81.0	0	< 0.001

The results are shown as the median values (interquartile range) or percentage. The Kruskal–Wallis test or chi-square test was conducted to examine the differences in individual clinical parameters among quadrants between the three groups. The Steel test was used to compare the normo-albuminuria group with the other three groups.

*BMI* body mass index, *CCB* calcium channel blocker, *DBP* diastolic blood pressure, *GMI* glucose management indicator, *GLP-1 RA* glucagon-like peptide-1 receptor agonist, *GRI* glycemia risk index, *HDL-chol* high-density lipoprotein cholesterol, *NDM* non-diabetes mellitus, *RAS* renin–angiotensin system, *SBP* systolic blood pressure, *SGLT2* sodium–glucose cotransporter 2, *T2DM* type 2 diabetes mellitus, *TAR* time above range, *TBR* time below range, *T-chol* total cholesterol, *TIR* time in range, and *UA* uric acid

**P < 0.01, *P < 0.05

### Differences in renal function and urinary biomarkers based on DKD stages

**Fig. 1:**
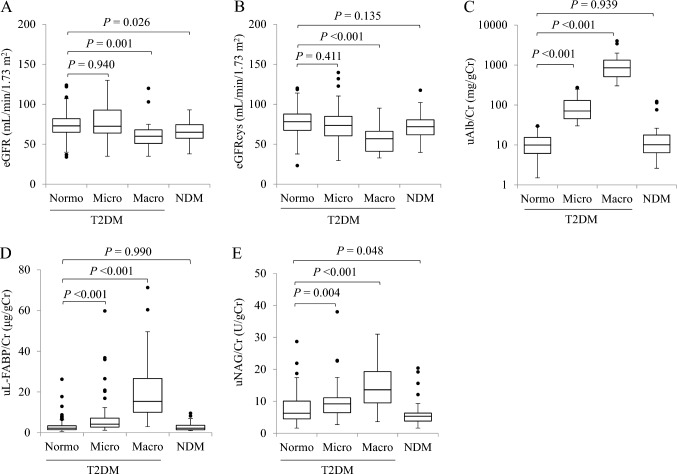
Relationship between the urinary albumin excretion rate and renal function and urinary biomarkers. **A** Estimated glomerular filtration rate (eGFR), **B** cystatin C with eGFR (eGFRcys), **C** urinary albumin-to-creatinine ratio (uAlb/Cr), **D** urinary liver-type fatty acid-binding protein-to-creatinine ratio (uL-FABP/Cr), and **E** urinary *N*-acetyl-β-d-glucosaminidase-to-creatinine ratio (uNAG/Cr). Data are shown in box and whisker plots. The black dots are outliers. Macro, macroalbuminuria; Micro, microalbuminuria; NDM, non-diabetes mellitus; Normo: normo-albuminuria; T2DM, type 2 diabetes mellitus

The results of eGFR, eGFRcys, and urinary biomarkers in each group are shown in Fig. 1. The eGFR was not significantly different between the normo group and the micro group [73.0 (65.0–82.0) vs*.* 72.5 (64.0–92.8) mL/min/1.73 m^2^, *P* = 0.940]; however, the rates in the macro group [60.0 (51.0–68.0) mL/min/1.73 m^2^, *P* = 0.001] and NDM group [65.0 (57.5–74.5) mL/min/1.73 m^2^, *P* = 0.026] were significantly lower than those in the normo group. For eGFRcys, only the macro group had significantly lower eGFRcys than the normo group did [56.9 (41.2–66.2) vs*.* 78.3 (67.5–87.8) mL/min/1.73 m^2^, *P* < 0.001].

The uAlb/Cr in the normo group was not significantly different from that in the NDM group [10.0 (6.1–15.4) vs*.* 10.1 (6.4–17.7) mg/gCr, *P* = 0.939] but increased significantly with a worsening DKD stage. The uL-FABP/Cr was not significantly different between the normo group and the NDM group [2.2 (1.4–3.4) vs*.* 2.2 (1.4–3.6) μg/gCr, *P* = 0.990], whereas the micro group [4.2 (2.7–7.1) μg/gCr, *P* < 0.001] and the macro group [15.4 (10.0–26.6) μg/gCr, *P* < 0.001] had significantly higher uL-FABP/Cr than the normo group. The uNAG/Cr was significantly lower in the NDM group [5.3 (3.8–6.3) U/gCr, *P* = 0.048] but was significantly higher in the micro [9.2 (6.4–11.1) U/gCr,* P* = 0.004] and macro [13.6 (9.5–19.3) U/gCr, *P* < 0.001] groups than in the normo group [6.3 (4.5–10.1) U/gCr].

### Differences in renal function and urinary biomarkers based on TIR

**Fig. 2:**
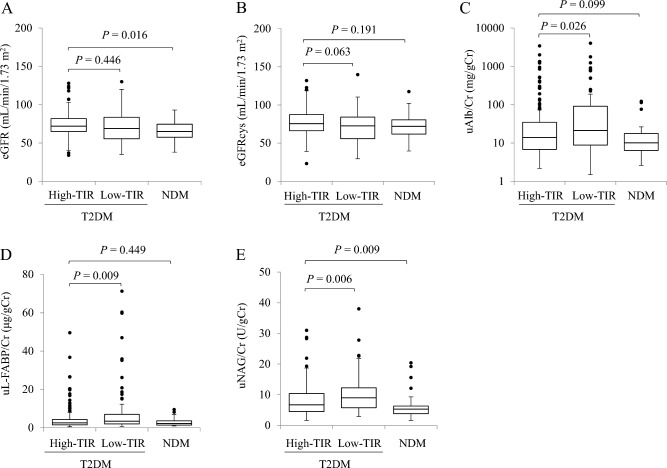
Relationship between the time in range (TIR) and renal function and urinary biomarkers. **A** Estimated glomerular filtration rate (eGFR), **B** cystatin C with eGFR (eGFRcys), **C** urinary albumin-to-creatinine ratio (uAlb/Cr), **D** urinary liver-type fatty acid-binding protein-to-creatinine ratio (uL-FABP/Cr), and **E** urinary *N*-acetyl-β-d-glucosaminidase-to-creatinine ratio (uNAG/Cr). Data are shown in box and whisker plots. The black dots are outliers. Macro, macroalbuminuria; Micro, microalbuminuria; Normo, normo-albuminuria; NDM, non-diabetes mellitus; T2DM, type 2 diabetes mellitus

Patients with T2DM were divided into two groups based on TIR, and renal function and urinary biomarkers in the low-TIR and NDM groups were compared with those in the high-TIR group as controls (Table 2 and Fig. 2). No significant difference in eGFRcys was found between the low-TIR [72.6 (55.9–84.2) mL/min/1.73 m^2^, *P* = 0.063] and NDM [72.0 (61.9–80.6) mL/min/1.73 m^2^, *P* = 0.191] groups and the high-TIR group [75.6 (66.3–87.4) mL/min/1.73 m^2^]. The uAlb/Cr was significantly higher in the low-TIR group than in the high-TIR group [21.2 (8.8–90.7) vs*.* 13.9 (6.8–34.6) mg/gCr, *P* = 0.026], whereas the NDM group [10.1 (6.4–17.7) mg/gCr, *P* = 0.099] did not show significant difference from the high-TIR group. Similar results were obtained for the uL-FABP/Cr. The uNAG/Cr was significantly lower in the NDM group than in the high-TIR group [5.3 (3.8–6.3) vs*.* 6.7 (4.5–10.4) U/gCr, *P* = 0.009], whereas it was significantly higher in the low-TIR group [9.0 (5.8–12.3) U/gCr, *P* = 0.006] than in the high-TIR group.

**Table 2:** Differences in patients’ backgrounds based on time in range (TIR)

	TIR in T2DM		NDM	*P*
	High TIR (*N* = 166)	Low TIR (*N* = 79)	(*N* = 39)	
Male, %	69.3	65.8	46.2	0.024
Age, years	68 (62–72)	69 (62–73)	71 (68–74)**	0.007
Duration of T2DM, years	11.5 (6.0–20.0)	17.0 (9.0–27.0)**	0 (0–0)**	0.001
BMI, kg/m^2^	24.2 (22.6–26.2)	24.0 (21.7–26.6)	23.9 (22.1–26.1)	0.780
SBP, mmHg	127.0 (116.0–138.0)	130.0 (116.0–141.0)	130.0 (121.5–139.0)	0.545
DBP, mmHg	76.0 (69.0–82.8)	75.0 (70.0–83.5)	78.0 (70.5–83.0)	0.682
UA, mg/dL	5.3 (4.6–6.2)	5.1 (4.3–6.1)	5.2 (4.8–6.1)	0.328
HbA1c, % [mmol/mol]	6.8 (6.5–7.3) [50]	7.7 (7.4–8.3) [60]**	5.9 (5.7–6.1) [40]**	< 0.001
GMI, %	6.4 (6.2–6.7)	7.5 (7.2–8.0)**	5.9 (5.7–6.0)**	< 0.001
TIR, %	85.7 (73.3–90.7)	54.6 (40.3–64.7)**	93.8 (91.1–97.4)**	< 0.001
Current smoker, %	21.7	15.2	5.1	0.041
Hypertension, %	66.9	73.4	74.4	0.461
Dyslipidemia, %	71.7	87.3	79.5	0.023
T-chol, mg/dL	177.5 (154.0–199.8)	186.0 (158.5–214.5)	188.0 (174.0–200.5)	0.098
HDL-chol, mg/dL	56.5 (48.0–67.8)	55.0 (44.0–74.5)	60.0 (53.0–71.0)	0.336
CCBs, %	39.2	41.8	48.7	0.547
RAS inhibitors, %	44.6	58.2	28.2	0.007
Statins, %	54.8	62.0	61.5	0.495
SGLT2 inhibitors, %	27.7	30.4	0	< 0.001
GLP-1 RAs, %	11.4	17.7	0	0.018
Other T2DM medicines, %	77.7	88.6	0	< 0.001

The results are shown as the median values (interquartile range) or percentage. The Kruskal–Wallis test or chi-square test was conducted to examine the differences in individual clinical parameters among quadrants between the three groups. The Steel test was used to compare the high-TIR (TIR ≥ 70.0%) group with the other two groups.

*BMI* body mass index, *CCB* calcium channel blocker, *DBP* diastolic blood pressure *GMI* glucose management indicator, *GLP-1 RA* glucagon-like peptide-1 receptor agonist, *HDL-chol* high-density lipoprotein cholesterol, *NDM* non-diabetes mellitus, *RAS* renin–angiotensin system, *SGLT2* sodium–glucose cotransporter 2, *T2DM* type 2 diabetes mellitus, and *T-chol* total cholesterol

**P < 0.01, *P < 0.05

### Relationship between urinary biomarkers and each glycemic control index

The relationship between the CGM-derived glycemic control indices and uAlb/Cr is shown in Table 3. In the univariate analysis, the uAlb/Cr was significantly associated with TIR (crude odds ratio (OR) 0.980, 95% confidence interval (CI) 0.967–0.992, *P* = 0.002), TIT (crude OR 0.981, 95% CI 0.970–0.992, *P* < 0.001), TAR (crude OR 1.021, 95% CI 1.009–1.034, *P* < 0.001), and SD (crude OR 1.023, 95% CI 1.003–1.042, *P* = 0.020). In addition, a significant association was found between the uAlb/Cr and GRI, an indicator of risk for hyperglycemia and hypoglycemia (crude OR 1.013, 95% CI 1.003–1.024, *P* = 0.012). On the contrary, no significant association was found between the uAlb/Cr and TBR (crude OR 0.974, 95% CI 0.929–1.021, *P* = 0.275) and CV (crude OR 0.998, 95% CI 0.960–1.036, *P* = 0.907). In Model 2, which was adjusted for multiple factors, such as eGFR, duration of diabetes, sex, age, BMI, smoking status, and presence of hypertension and dyslipidemia, a significant association was found between uAlb/Cr and each of the CGM-derived glycemic control indices, such as TIR (OR 0.985, 95% CI 0.971–0.998, *P* = 0.029), TAR (OR 1.015, 95% CI 1.002–1.029, *P* = 0.028), and GRI (OR 1.012, 95% CI 1.001–1.024, *P* = 0.038). Although GMI (OR 1.485, 95% CI 1.067–2.067, *P* = 0.019) was significantly associated with uAlb/Cr, no significant association was observed between HbA1c (OR 1.127, 95% CI 0.807–1.574, *P* = 0.483) and uAlb/Cr.

**Table 3:** Relationship between urinary albumin-to-creatinine ratio (uAlb/Cr) and glycemic control status

			Model 1		Model 2	
	Crude OR (95% CI)	*P*	OR (95% CI)	*P*	OR (95% CI)	*P*
UAlb/Cr						
HbA1c	1.424 (1.073–1.889)	0.014	1.125 (0.806–1.571)	0.490	1.127 (0.807–1.574)	0.483
GMI	1.710 (1.259–2.324)	0.014	1.484 (1.066–2.067)	0.019	1.485 (1.067–2.067)	0.019
TIR	0.980 (0.967–0.992)	0.002	0.985 (0.971–0.998)	0.029	0.985 (0.971–0.998)	0.029
TIT	0.981 (0.970–0.992)	< 0.001	0.986 (0.974–0.998)	0.021	0.986 (0.974–0.998)	0.021
TAR	1.021 (1.009–1.034)	< 0.001	1.015 (1.002–1.029)	0.028	1.015 (1.002–1.029)	0.028
TBR	0.974 (0.929–1.021)	0.275	0.989 (0.938–1.042)	0.677	0.988 (0.937–1.042)	0.665
SD	1.023 (1.003–1.042)	0.020	1.023 (1.000–1.046)	0.052	1.022 (1.000–1.046)	0.053
CV	0.998 (0.960–1.036)	0.907	1.007 (0.961–1.056)	0.760	1.007 (0.960–1.056)	0.776
GRI	1.013 (1.003–1.024)	0.012	1.012 (1.001–1.024)	0.037	1.012 (1.001–1.024)	0.038

In model 1, ordinal logistic regression analysis was performed with different stages of uAlb/Cr as the objective variable and each glycemic control index as explanatory variables, adjusted for duration of diabetes, sex, age, body mass index, smoking status, and presence of hypertension and dyslipidemia. Model 2 was adjusted for eGFR in addition to the factors in model 1

*CI* confidence interval, *CV* coefficient of variation, *GMI* glucose management indicator, *GRI* glycemia risk index, *OR* odds ratio, *SD* standard deviation, *TAR* time above range, *TBR* time below range, *TIR* time in range, *TIT* time in tight range

The relationship between the uL-FABP/Cr and uNAG/Cr and each glycemic control index is shown in Table 4. In the univariate analysis, the uL-FABP/Cr was significantly associated with TIR (crude OR 0.977, 95% CI 0.961–0.993, *P* = 0.004), TIT (crude OR 0.982, 95% CI 0.967–0.996, *P* = 0.014), TAR (crude OR 1.022, 95% CI 1.006–1.038, *P* = 0.006), and GRI (Crude OR 1.018, 95% CI 1.004–1.031, *P* = 0.010). Similar results were obtained in Model 1, which was adjusted for multivariate factors. However, the logistic regression analysis adjusted for uAlb/Cr and eGFR showed no significant association between uL-FABP/Cr and each CGM-derived glycemic control index. On the contrary, the multivariate analysis indicated that uNAG/Cr was significantly associated not only with TIR (OR 0.973, 95% CI 0.957–0.989, *P* = 0.001) and TIT (OR 0.981, 95% CI 0.968–0.993, *P* = 0.003) but also with TAR (OR 1.024, 95% CI 1.008–1.040, *P* = 0.004) and GRI (OR 1.019, 95% CI 1.006–1.032, *P* = 0.004).

**Table 4:** Relationship between urinary biomarkers of tubular injury and glycemic control status

			Model 1		Model 2	
	Crude OR (95% CI)	*P*	OR (95% CI)	*P*	OR (95% CI)	*P*
uL-FABP/Cr						
HbA1c	1.462 (1.000–2.137)	0.050	1.399 (0.902–2.169)	0.134	1.556 (0.825–2.935)	0.172
GMI	1.646 (1.112–2.425)	0.013	1.613 (1.052–2.474)	0.028	1.187 (0.612–2.304)	0.611
TIR	0.977 (0.961–0.993)	0.004	0.978 (0.961–0.996)	0.015	0.990 (0.963–1.018)	0.478
TIT	0.982 (0.967–0.996)	0.014	0.983 (0.967–0.999)	0.039	0.995 (0.971–1.019)	0.690
TAR	1.022 (1.006–1.038)	0.006	1.021 (1.004–1.039)	0.017	1.010 (0.984–1.037)	0.463
TBR	0.997 (0.941–1.056)	0.913	0.995 (0.931–1.063)	0.873	0.994 (0.923–1.071)	0.881
SD	1.034 (1.009–1.061)	0.008	1.030 (1.001–1.060)	0.044	1.014 (0.974–1.056)	0.493
CV	1.035 (0.986–1.086)	0.164	1.021 (0.961–1.084)	0.508	1.019 (0.948–1.096)	0.606
GRI	1.018 (1.004–1.031)	0.010	1.017 (1.002–1.033)	0.022	1.006 (0.984–1.029)	0.580
uNAG/Cr						
HbA1c	1.713 (1.280–2.292)	< 0.001	1.663 (1.195–2.314)	0.003	1.625 (1.159–2.279)	0.005
GMI	1.780 (1.280–2.475)	< 0.001	1.751 (1.219–2.516)	0.002	1.019 (1.006–1.032)	0.004
TIR	0.972 (0.958–0.987)	< 0.001	0.971 (0.956–0.987)	< 0.001	0.973 (0.957–0.989)	0.001
TIT	0.979 (0.968–0.990)	< 0.001	0.978 (0.966–0.991)	< 0.001	0.981 (0.968–0.993)	0.003
TAR	1.026 (1.012–1.040)	< 0.001	1.026 (1.010–1.042)	0.001	1.024 (1.008–1.040)	0.004
TBR	0.999 (0.963–1.036)	0.946	1.011 (0.971–1.052)	0.606	1.014 (0.973–1.057)	0.503
SD	1.038 (1.017–1.060)	< 0.001	1.042 (1.017–1.067)	< 0.001	1.038 (1.013–1.064)	0.002
CV	1.025 (0.989–1.064)	0.179	1.030 (0.989–1.073)	0.152	1.032 (0.990–1.076)	0.140
GRI	1.019 (1.008–1.031)	0.001	1.021 (1.008–1.033)	0.001	1.019 (1.006–1.032)	0.004

In model 1, logistic regression analysis was performed with urinary liver-type fatty acid-binding protein-to-creatinine ratio (uL-FABP/Cr) or urinary *N*-acetyl-β-d-glucosaminidase-to-creatinine ratio (uNAG/Cr) positive as the objective variable and each glycemic control index as explanatory variables adjusted for duration of diabetes, sex, age, body mass index, smoking status, and presence of hypertension and dyslipidemia. Model 2 was adjusted for eGFR and urinary albumin-to-creatinine ratio in model 1

*CI* confidence interval, *CV* coefficient of variation, *GMI* glucose management indicator, *GRI* glycemia risk index, *OR* odds ratio, *SD* standard deviation, *TAR* time above range, *TBR* time below range, *TIR* time in range, and *TIT* time in tight range

## Discussion

The findings of this study indicated that uL-FABP/Cr is significantly elevated in the early stages of DKD with microalbuminuria in patients with T2DM. On the contrary, uNAG/Cr was significantly higher even in patients with T2DM and normo-albuminuria than in those with NDM, and uNAG/Cr increased with worsening DKD stage. Furthermore, not only uAlb/Cr but also uNAG/Cr were significantly associated with CGM-derived glycemic control indices, such as TIR, TIT, and GRI, independent of renal function and the presence of hypertension.

Consistent with previous reports, the results of this study indicated that uAlb/Cr, used as a marker of glomerular damage, was associated with CGM-derived TIR and GRI [19, 20, 32, 33]. As both TIR and GRI are considered indicators of the quality of glycemic control [28], glomerular dysfunction may be associated with poorer quality of glycemic control. On the other hand, several studies have suggested that proximal tubular damage is important in the DKD onset and progression and that tubular damage precedes glomerular damage [8–10]. Several renal proximal tubular injury markers have been detected in the urine of patients with T2DM but without overt glomerular damage. For example, uL-FABP/Cr and uNAG/Cr have been reported to be significantly higher in T2DM with normo-albuminuria than in NDM [11–18]. The results of this study showed that the normo-albuminuria group had a significantly higher uNAG/Cr than the NDM group, whereas uL-FABP/Cr did not differ significantly between the two groups. The relationship between increased uL-FABP/Cr and DKD severity varies between studies [11–13, 34]; however, uNAG/Cr precedes increased urinary albumin excretion [14–18, 35].

UL-FABP/Cr and uNAG/Cr are urinary biomarkers of tubular damage. L-FABP is located in the cytoplasm of human renal proximal tubular cells and is engaged in free fatty acid metabolism [34]. UL-FABP/Cr increases with excessive stress on the tubules, including urinary protein excretion, hypertension, tubular ischemia, and oxidative stress [36–38]. In addition, uL-FABP/Cr decreases along with uAlb/Cr using RAS inhibitors or statins [37–39]. This study showed no significant association between uL-FABP/Cr and CGM-derived glycemic control indices in a multivariate analysis adjusted for uAlb/Cr and eGFR. Thus, uL-FABP/Cr may be related to factors, such as the presence of albuminuria or eGFR, rather than glycemic control in patients with T2DM.

In contrast to uL-FABP/Cr, this study showed that uNAG/Cr was associated with CGM-derived glycemic control indices, such as TIR and GRI, independent of the uAlb/Cr and eGFR levels. NAG is one of the glycoproteinases contained in intracellular lysosomes, and its urinary excretion is increased in response to the injury of lysosomes in tubular epithelial cells [11]. Zheng HJ et al. reported that sustained hyperglycemic exposure may impair lysosomal function [40]. Furthermore, a positive correlation was found between uNAG/Cr and blood glucose levels and uNAG/Cr increases with high glucose load [41–43]. Thus, among the urinary biomarkers, uNAG/Cr is assumed to be more sensitive to hyperglycemia [44, 45]. In fact, the findings of this study indicated that TAR and GRI were associated with uNAG/Cr. In addition, the results also showed that TIT was associated with uNAG/Cr. Because uNAG/Cr decreases with lowering blood glucose levels [46, 47], increasing the percentage of time spent in the 70–140 mg/dL range might decrease uNAG/Cr. Although further validation is needed to elucidate the pathogenesis mechanism described above, this study is valuable as the first to show an association between newly developed glycemic control indices such as GRI and TIR and tubular damage markers in patients with DKD.

This study had some limitations. First, it was a cross-sectional study. Therefore, long-term prospective studies on the relationship between urinary biomarkers and DKD progression are needed. In addition, this study measured uAlb/Cr, uL-FABP/Cr, and uNAG/Cr as urinary biomarkers. Other tubular damage markers include neutrophil gelatinase-associated lipocalin, kidney injury molecule-1, and β2-microglobulin [48]. Therefore, studies including these urinary biomarkers might be necessary.

## Conclusions

The results of this study indicate that urinary NAG, which is largely located in the proximal tubule cells and is a leakage marker of epithelial injury, is significantly high even in patients with T2DM in the normo-albuminuria stage. Furthermore, this study showed for the first time that uNAG/Cr was associated with the CGM-derived glycemic control indices, such as TIR and GRI, independent of albuminuria and eGFR levels.

## Data Availability

The individual deidentified participant data will be shared upon reasonable request to the corresponding author.

## Abbreviations

DN: Diabetic nephropathy
DM: Diabetes mellitus
eGFR: Estimated glomerular filtration rate
uAlb/Cr: Urinary albumin-to-creatinine ratio
DKD: Diabetic kidney disease
uL-FABP/Cr: Urinary liver-type fatty acid-binding protein-to-creatinine ratio
uNAG/Cr: Urinary *N*-acetyl-β-d-glucosaminidase-to-creatinine ratio
TIR: Time in range
CGM: Continuous glucose monitoring
T2DM: Type 2 diabetes mellitus
NDM: Non-diabetes mellitus
HDHCC: Hyogo Diabetes Hypoglycemia Cognition Complications
TIT: Time in the tight range
TAR: Time above range
TBR: Time below range
CV: Coefficient of variation
SD: Standard deviation
GMI: Glucose management indicator
GRI: Glycemia risk index
eGFRcys: Cystatin C with eGFR
OR: Odds ratio
CI: Confidence interval
